# Central versus peripheral veno-arterial extracorporeal membrane oxygenation during lung transplantation: a systematic review and meta-analysis

**DOI:** 10.1016/j.jhlto.2026.100502

**Published:** 2026-01-29

**Authors:** Lucas Monteiro Delgado, Júlia Luíza do Sacramento Silva, Rachid Eduardo Noleto da Nobrega Oliveira, Tulio Caldonazo, Felipe S. Passos, Erlon de Avila Carvalho

**Affiliations:** a Hospital das Clínicas, Federal University of Minas Gerais, Belo Horizonte, Minas Gerais, Brazil; b Faculdade de Ciências Médicas de Minas Gerais, Belo Horizonte, Minas Gerais, Brazil; c Department of Thoracic Surgery, Barretos Cancer Hospital, Barretos, São Paulo, Brazil; d Brazilian Thoracic Surgery Research Group; e Department of Cardiothoracic Surgery, Jena University Hospital, Jena, Germany; f Department of Thoracic Surgery, Mater Dei Hospital, Salvador, Bahia, Brazil

**Keywords:** Lung transplantation, Veno-arterial ECMO, Central cannulation, Peripheral cannulation

## Abstract

**Introduction:**

The use of extracorporeal membrane oxygenation (ECMO) during lung transplantation has progressively expanded and, in many centers, replaced conventional cardiopulmonary bypass. However, it remains unclear whether central or peripheral veno-arterial (VA) ECMO provides superior postoperative outcomes. This study aimed to compare central VA-ECMO (cVA-ECMO) and peripheral VA-ECMO (pVA-ECMO) during lung transplantation, with a focus on survival, primary graft dysfunction grade 3 (PGD3), postoperative ECMO support, and postoperative morbidity.

**Methods:**

Three databases were assessed through November 2025. Five retrospective observational studies including 866 patients were included. Overall survival was analyzed using reconstructed individual patient data derived from Kaplan–Meier curves. Random-effects models were applied for all pooled analyses.

**Results:**

There was no significant difference in overall survival (HR 1.224, p=0.13) and in PGD3 at 72 h incidence (OR 1.55; p=0.26) between cVA-ECMO and pVA-ECMO. However, pVA-ECMO was associated with a higher requirement for postoperative ECMO use (OR 6.05; p=0.04), longer duration of extracorporeal support (MD +1.61 days; p=0.01), prolonged mechanical ventilation (MD +2.73 days; p<0.01), and longer intensive care unit length of stay (MD +4.05 days; p<0.01). The risk of limb ischemia requiring invasive treatment was significantly higher with pVA-ECMO (OR 4.94; p=0.001).

**Conclusion:**

Although survival and PGD3 incidence were comparable, pVA-ECMO was associated with greater postoperative morbidity and vascular complications. These findings should be interpreted with caution, and cannulation strategy should be individualized according to patient risk profile, surgical context, and center-specific expertise rather than favoring one approach uniformly.

## Introduction

Over the past 40 years, lung transplantation has become a viable treatment option for patients with various end-stage lung diseases,^1^ establishing itself as definitive therapy for patients with advanced lung diseases. Nevertheless, it remains a highly complex procedure, particularly during the intraoperative period, when abrupt changes in ventilatory mechanics and cardiac function can precipitate hemodynamic instability and other complications. In this setting, optimizing circulatory and respiratory support is essential to ensure operative safety.

Extracorporeal membrane oxygenation (ECMO) has become increasingly integrated into lung transplantation worldwide and, in many centers, has partially replaced conventional cardiopulmonary bypass due to its ability to provide effective cardiopulmonary support with improved perioperative outcomes.^2^ Beyond its established role as rescue support for intraoperative instability and for the management of primary graft dysfunction (PGD) in the early post operative period, ECMO is now applied across the entire transplant environment, including as a bridge to transplant, a bridge-to-decision for rapidly deteriorating patients, and as planned intraoperative support.^3^, ^4^, ^5^ Intraoperatively, ECMO provides stable oxygenation and hemodynamic control, attenuates the systemic inflammatory response associated with cardiopulmonary bypass, limits hemodilution and transfusion requirements, and protects the right ventricle in the setting of elevated pulmonary vascular load.^3^, ^5^

Two main cannulation strategies are used during intraoperative venoarterial (VA) ECMO: central and peripheral. Central VA-ECMO (cVA-ECMO), performed by direct cannulation of the right atrium and aorta, provides physiological anterograde flow, adequate venous drainage, and superior hemodynamic stability, though at the cost of greater surgical invasiveness. In contrast, peripheral VA-ECMO (pVA-ECMO), usually femoral, is less invasive, avoids direct manipulation of major thoracic structures, and facilitates rapid transitions to postoperative support. However, it may generate retrograde aortic flow, affect ventriculo-aortic interaction, and increase the risk of peripheral vascular complications.^1^, ^5^, ^6^, ^7^

Despite the widespread use of both strategies, there is no consensus regarding which modality offers better intraoperative support during lung transplantation. In this context, we conducted a systematic review and meta-analysis to compare the clinical, perioperative, and postoperative outcomes associated with cVA-ECMO versus pVA-ECMO during the intraoperative period of lung transplantation.

## Methods

This systematic review and meta-analysis followed the Cochrane Handbook for Systematic Reviews of Interventions and the Preferred Reporting Items for Systematic Reviews and Meta-Analysis (PRISMA) guidelines.^8^, ^9^ The study protocol was registered in the International Prospective Register of Systematic Reviews (PROSPERO, CRD420251236937).^10^

### Search strategy

A comprehensive literature search was performed on EMBASE, MEDLINE, and Cochrane Library databases from inception through November 2025. We also searched the references of the included studies and previous systematic reviews and meta-analyses aiming for the inclusion of additional studies.^11^ The search strategy for each database is detailed in Supplemental Table 1.

### Study selection

Two independent authors (L.M.D. and J.L.S.S.) initially imported the studies into Rayyan Software (Qatar Computing Research Institute, Qatar Foundation) for deduplication and screening. Discrepancies between reviewers were resolved through discussion and consensus. Titles and abstracts were reviewed against pre-defined inclusion and exclusion criteria.

### Eligibility criteria

Inclusion in this meta-analysis was restricted to studies meeting all of the following eligibility criteria: (1) randomized controlled trials (RCTs) or observational studies enrolling adult patients undergoing lung transplantation; (2) studies comparing cVA-ECMO with pVA-ECMO; and (3) reporting at least one relevant clinical outcome. Exclusion criteria included studies involving pediatric patients, studies without a clear distinction between cVA-ECMO and pVA-ECMO, studies using animal models, conference abstracts, case reports, studies with insufficient data, and non-comparative study designs. No restrictions were applied regarding the timing or strategy of ECMO initiation, and both preemptive and unplanned (rescue) intraoperative ECMO use were eligible for inclusion.

### Data extraction

Two authors (L.M.D. and J.L.S.S.) independently extracted data using a standardized form. The extracted variables included study characteristics (author, publication year, time frame, country, sample size, intervention and control groups), patient demographics (age, sex, underlying disease, type of transplant), donor characteristics (donor age, donor sex, smoking history, donation after circulatory death, and pre-procurement ventilation duration), and reported outcomes.

### Risk of bias assessment

Two independent reviewers (L.M.D and J.L.S.S.) assessed the Risk of Bias In Non-Randomized Studies of Interventions (ROBINS-I V2) tool.^12^ Disagreements were resolved through consensus. Publication bias could not be assessed adequately because the power of this test is insufficient to discriminate between chance and true funnel plot asymmetry when analyzing fewer than 10 studies.^13^

### Outcomes

The outcomes of interest were: (1) overall survival (OS); (2) primary graft dysfunction grade 3 at 72 h (PGD3 at 72 h); (3) postoperative ECMO use; (4) ECMO duration; (5) time until extubation (ventilation time); (6) intensive care unit length of stay (ICU LOS); (7) limb ischemia requiring invasive treatment; and (8) arterial dissection.

### Statistical analysis

Mean differences (MD) and odds ratios (ORs) with their 95% confidence intervals (CIs) were pooled for continuous and binary outcomes, respectively. Time-to-event data strategy was utilized for overall survival. A p-value < 0.05 was considered statistically significant for overall effect estimates. DerSimonian and Laird random-effects models were used for all outcomes.^14^ Between-study heterogeneity was assessed using the Cochran Q test and I^2^ statistics, and we considered a P value of <0.10 and an I^2^ >25% as significant for heterogeneity. For outcomes with significant heterogeneity, we performed leave-one-out sensitivity analysis to identify influential studies and their effect on the pooled estimates. The Cochrane Handbook for Systematic Reviews of Interventions was used for data handling and conversion.^8^ All statistical analyses were conducted using R statistical software (version 4.4.2; R Foundation for Statistical Computing, Vienna, Austria) and Stata (version 17.0; StataCorp LLC, College Station, TX, USA).

### Individual patient survival data meta-analysis

We used the methods described by Guyot et al. to reconstruct individual patient data (IPD) from the Kaplan-Meier curves of all eligible studies for overall survival outcome.^15^ All Kaplan–Meier survival curves were pre-processed and digitized using WebPlotDigitizer, so that the values reflecting specific timepoints with their corresponding survival information could be extracted. When available, numbers at risk were also collected.^16^

The Kaplan–Meier (KM) method was used to estimate overall survival.^17^ Between-group differences were assessed using Cox proportional hazards regression.^18^ The proportional hazards assumption was evaluated by inspecting scaled Schoenfeld residuals (Schoenfeld, 1982) and using the Grambsch–Therneau test,^19^, ^20^ as well as by examining log–log survival plots and comparing predicted versus observed survival functions.^21^, ^22^ Survival curves were plotted using the Kaplan–Meier product-limit method, and hazard ratios (HRs) with 95% confidence intervals were reported for each group.

## Results

### Study selection

**Figure 1:**
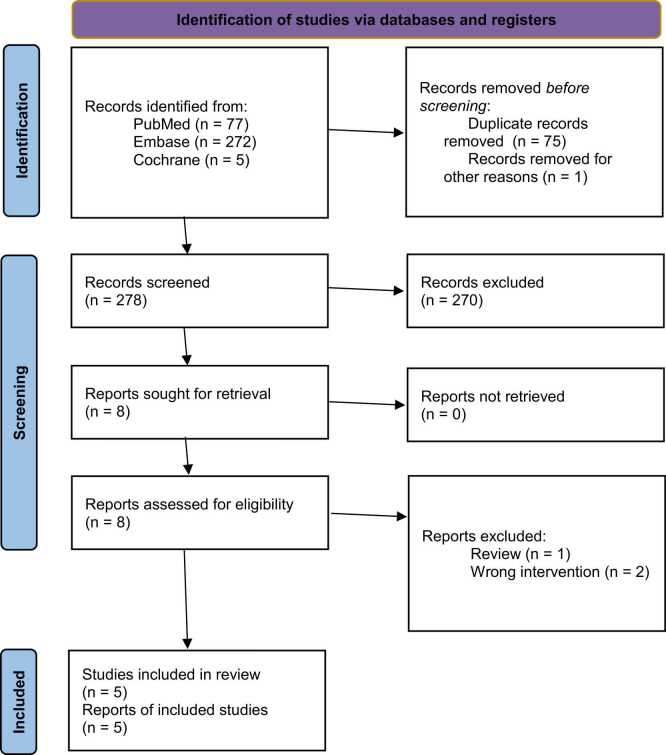
Preferred Reporting Items for Systematic Reviews and Meta-Analyses (PRISMA) flow diagram.

As detailed in Figure 1, the initial search identified 354 results. After removal of duplicate records and assessment of the studies based on title and abstract, 8 full-text studies remained for full review according to prespecified criteria. Of these, five retrospective observational studies, spanning from 2018 to 2025, were included.^23^, ^24^, ^25^, ^26^, ^27^ Three studies were excluded during the full-text screening stage: the study by Toubat et al.^28^ was an institutional algorithm proposal for intraoperative ECMO management, the study by Patrick et al.^29^ did not compare ECMO modalities, and the study by Schoeberl et al.^30^ included VV-ECMO in the cohort. Details on excluded articles in the full-text assessment triage are provided on Supplemental Table 2.

### Study and patient characteristics

Five retrospective observational studies were included, encompassing a total of 866 lung transplant recipients managed with ECMO support. Among these, 428 (48.3%) patients underwent lung transplantation with cVA-ECMO and 448 (51.7%) were managed with pVA-ECMO. Across studies, the number of patients ranged from 32 to 501. The study periods spanned from 2009 to 2024 and included multicenter cohorts from the United States and Europe as well as single-center experiences from France, China, and Poland. The age at transplantation ranged from 42 to 57 years, and the proportion of female recipients varied between approximately 25% and 54%. Femoral–femoral (F–F) cannulation was used in three studies, femoral–axillary (F–A) in one, and a mixed F–F/A–F configuration in one. The main demographic and clinical characteristics are summarized in Table 1. Donor characteristics are provided in Supplemental Table 3.

**Table 1:** Study and Recipient Characteristics across Included Studies in the Meta-analysis

Study	Time Frame	Country	Sample Size, n (%)	Age, Median or Mean	Female, n (%)	Type of Incision, Central/ Peripheral, n (%)	Primary Diagnosis, Central/ Peripheral, n (%)
Fernandez, 2025	2016-2024	Multicentric; USA, Europe	Central: 315 (62.9) Peripheral: 186 (37.1)	Central: 57† Peripheral: 53†	Central: 138 (43.8) Peripheral: 86 (46.2)	Clamshell: 293 (93)/ 30 (16.1) Bilateral Thoracotomy: 17 (5.4)/ 155 (83.3) Sternotomy: 5 (1.6)/ 1 (0.5)	RLD: 197 (62.5)/ 108 (58.1) OLD: 85 (27)/ 41 (22) PVD: 22 (7)/ 15 (8.1) CF: 11 (3.5)/ 22 (11.8)
Glorion, 2018	2011-2016	France	Central: 54 (52.4) Peripheral: 49 (47.6)	Central: 50.5* Peripheral: 40*	Central: 29 (53.7) Peripheral: 25 (51)	Clamshell: 29 (53)/ 13 (27) Bilateral Thoracotomy: 25 (47)/ 36 (73) Sternotomy: 0 (0)/ 0 (0)	RLD: 14 (25.9)/ 11 (22.4) OLD: 9 (16.7)/ 6 (12.2) PVD: 26 (48.1)/ 30 (61.2) CF: 0 (0)/ 0 (0)
Li, 2024	2015-2021	China	Central: 30 (27.8) Peripheral: 78 (72.2)	Central: 56* Peripheral: 58*	Central: 12 (40) Peripheral: 20 (25.6)	NR	RLD: 22 (73.3)/ 59 (75.6) OLD: 11 (36.7)/ 19 (24.4) PVD: 0 (0)/ 0 (0) CF: 0 (0)/ 0 (0)
Ruszel, 2021	2009-2020	Poland	Central: 18 (56.3) Peripheral: 14 (43.7)	Central: 42† Peripheral: 49†	Central: 8 (44.4) Peripheral: 5 (35.7)	NR	RLD: 9 (50)/ 3 (21.4) OLD: 1 (5.6)/ 6 (42.9) PVD: 0 (0)/ 2 (14.3) CF: 7 (38.9)/ 2 (14.3)
Wu, 2025	2019-2023	China	Central: 31 (25.4) Peripheral: 91 (74.6)	Central: 57* Peripheral: 54*	Central: 9 (29.0) Peripheral: 25 (27.5)	Clamshell: 26 (83.9)/ 12 (13.2) Bilateral Thoracotomy: 5 (16.1)/ 79 (86.8) Sternotomy: 0 (0)/ 0 (0)	RLD: 20 (64.5)/ 59 (64.8) OLD: 8 (25.8)/ 20 (22) PVD: 0 (0)/ 0 (0) CF: 0 (0)/ 0 (0)

*Median; †Mean; CF: Cystic fibrosis; NR: Not reported; OLD: Obstructive lung disease; PVD: Pulmonary vascular disease; RLD: Restrictive lung disease

**Table 2:** Summary of Outcomes

Outcome	Number of Studies	Number of Patients	Effect Estimate, Random-effects Model (95% CI, p-value)
Overall survival	5	866	HR 1.224; 95% CI 0.94 to 1.59; p=0.135
Primary graft dysfunction grade 3 at 72 h	4	834	OR 1.55; 95% CI 0.72 to 3.31; p=0.26
Postoperative ECMO requirement	3	726	OR 6.05; 95% CI 1.10 to 33.24; p=0.04
ECMO duration (days)	3	333	MD 1.61; 95% CI 0.36 to 2.86; p=0.01
Ventilation time (days)	3	333	MD 2.73; 95% CI 1.52 to 3.95; p<0.01
ICU length of stay (days)	3	333	MD 4.05; 95% CI 2.45 to 5.64; p<0.01
Limb ischemia requiring invasive treatment	3	726	OR 4.94; 95% CI 1.96 to 12.45; p=0.01
Arterial dissection	3	712	OR 1.26; 95% CI 0.26 to 6.15; p=0.78

CI: confidence interval; ECMO: extracorporeal membrane oxygenation; ICU: intensive care unit; MD: mean difference; OR: odds ratio; OS: overall survival; PGD3: Primary graft dysfunction grade 3.

### Outcomes

#### Overall survival

**Figure 2:**
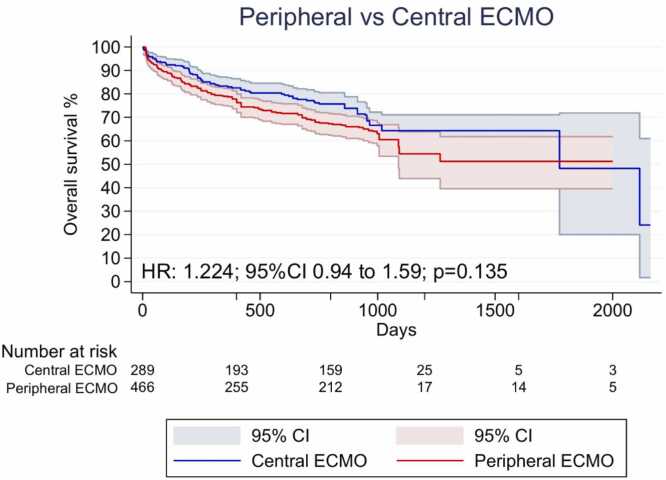
Overall survival for patients undergoing peripheral versus central VA-ECMO during lung transplantation. CI = confidence interval; HR = hazard ratio; VA-ECMO = venoarterial extracorporeal membrane oxygenation.

Overall, five KM curves were digitized, processed and reconstructed. There was no significant difference regarding OS between pVA-ECMO and cVA-ECMO support (HR 1.224; 95%CI 0.94 to 1.59; p=0.135; Figure 2).

The same tendency was detected when addressing the individual HRs of the studies in the two-stage meta-analysis, which showed no significant difference in survival between pVA-ECMO and cVA-ECMO support (HR 1.11; 95%CI 0.85 to 1.46; p=0.44; I²=0.00%; Supplemental Figure 1).

#### Primary graft dysfunction

**Figure 3:**
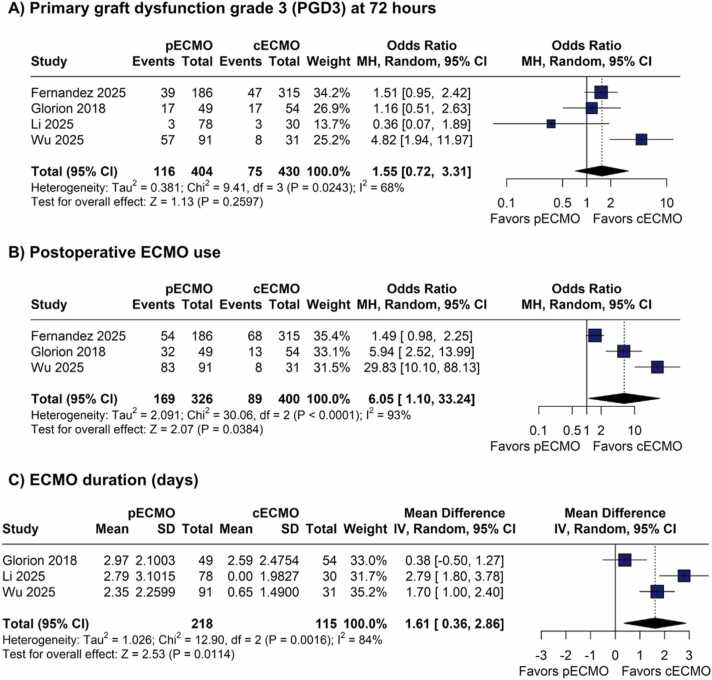
Forest plots comparing peripheral versus central VA-ECMO during lung transplantation. (A) Grade 3 primary graft dysfunction (PGD3) at 72 h. (B) Postoperative ECMO use. (C) ECMO duration (days). CI = confidence interval; MD = mean difference; OR = odds ratio; VA-ECMO = venoarterial extracorporeal membrane oxygenation.

There were no significant differences between groups for PGD3 at 72 h (OR 1.55; 95% CI 0.72 to 3.31; p=0.26; I²=68%; Figure 3A).

#### Postoperative ECMO use and ECMO duration

Regarding postoperative ECMO use (OR 6.05; 95% CI 1.10 to 33.24; p=0.04; I²=93%; Figure 3B) and ECMO duration (MD 1.61 days; 95% CI 0.36 to 2.86; p=0.01; I²=84%; Figure 3C), both were significantly higher in the pVA-ECMO group.

#### Time until extubation and ICU LOS

**Figure 4:**
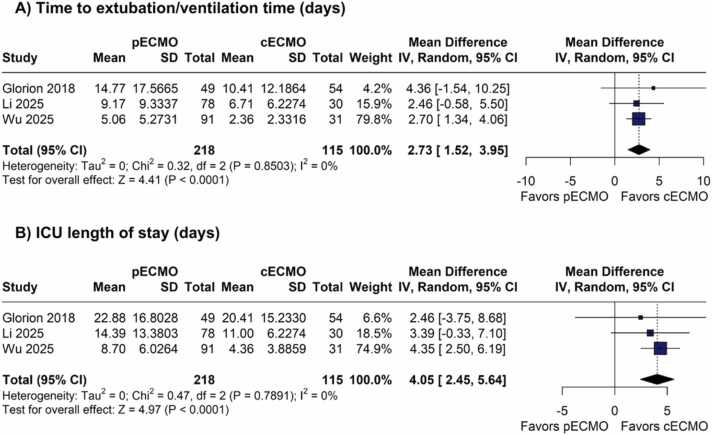
Forest plots comparing postoperative recovery outcomes between peripheral and central VA-ECMO during lung transplantation. (A) Mechanical ventilation duration (days). (B) Intensive care unit length of stay (days). CI = confidence interval; MD = mean difference; VA-ECMO = venoarterial extracorporeal membrane oxygenation.

Ventilation time (MD 2.73 days; 95% CI 1.52 to 3.95; p<0.01; I²=0%; Figure 4A) and ICU LOS (MD 4.05 days; 95% CI 2.45 to 5.64; p<0.01; I²=0%; Figure 4B) were significantly higher in the pVA-ECMO group.

#### Cannulation complications

**Figure 5:**
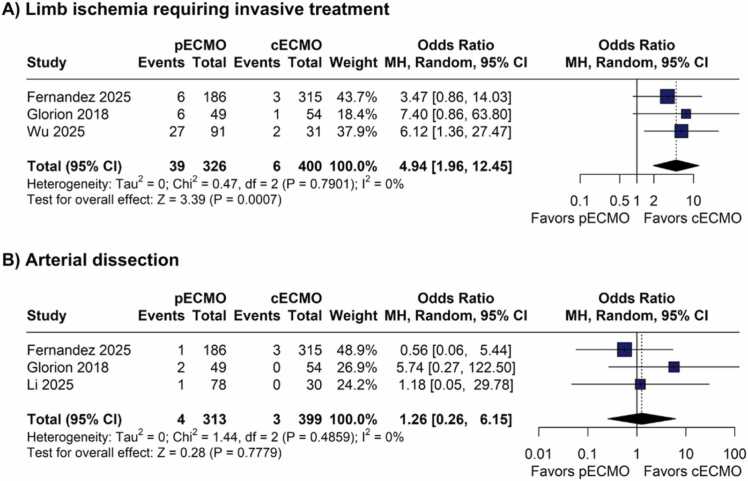
Forest plots comparing cannulation-related complications between peripheral and central VA-ECMO during lung transplantation. (A) Limb ischemia requiring invasive intervention. (B) Arterial dissection. CI = confidence interval; OR = odds ratio; VA-ECMO = venoarterial extracorporeal membrane oxygenation.

Limb ischemia requiring invasive treatment was significantly higher in the pVA-ECMO group (OR 4.94; 95% CI 1.96 to 12.45; p=0.01; I²=0%; Figure 5A). However, there were no significant differences between groups for arterial dissection (OR 1.26; 95% CI 0.26 to 6.15; p=0.78; I²=0%; Figure 5B).

#### Sensitivity analysis

Across the outcomes with high heterogeneity, the leave-one-out analysis showed that exclusion of any single study consistently reduced between-study heterogeneity. However, instability was observed for postoperative ECMO duration: removal of the studies by Li et al.^25^ or by Wu et al.^27^ shifted the pooled effect from favoring cVA-ECMO to a non-significant result. A similar pattern was observed for postoperative ECMO use, in which the exclusion of the studies by Glorion et al.^24^ or by Wu et al.^27^ altered the direction of the effect. These findings likely reflect the limited number of available studies, whereby omitting a single trial exerts a disproportionate influence on the pooled estimates. Detailed leave-one-out analysis are presented in Supplementary Figures 2–4.

#### Risk of bias assessment

Supplementary Figure 5 summarizes the overall risk-of-bias judgments across the included studies, showing that most were rated at a moderate level of concern. These assessments reflect the observational design and potential for residual confounding, although key domains such as intervention classification and adherence were consistently well evaluated. Supplementary Table 4 provides detailed rationales supporting each domain-specific judgment.

## Discussion

In this systematic review and meta-analysis of five studies including 866 patients, we comprehensively analyzed the outcomes of cVA-ECMO versus pVA-ECMO during the intraoperative period of lung transplantation. Our main findings were as follows: (I) there was no significant difference in overall survival between cVA-ECMO and pVA-ECMO support in lung transplantation; (II) there was no significant difference in the incidence of PGD3 at 72 h; (III) patients supported with pVA-ECMO had a significantly higher postoperative ECMO use and longer ECMO duration; (IV) pVA-ECMO was associated with longer mechanical ventilation time and ICU LOS; and (V) limb ischemia requiring invasive treatment was significantly more frequent in the pVA-ECMO group.

Our findings are consistent with the growing body of evidence supporting intraoperative ECMO as the preferred circulatory support strategy during lung transplantation, while refining our understanding of how cannulation configuration impacts postoperative outcomes. Recent systematic reviews and large observational studies have consistently demonstrated that ECMO, compared to conventional cardiopulmonary bypass, is associated with a reduced systemic inflammatory response, lower transfusion requirements, better right ventricular protection, and favorable early postoperative outcomes, particularly in high-risk recipients with pulmonary hypertension or hemodynamic instability.^2^, ^5^, ^6^, ^7^ As ECMO has become widely adopted as routine intraoperative support, the clinical focus has progressively shifted from the indication for ECMO use to optimization of its technical configuration.

Central and peripheral VA-ECMO differ in cannulation strategy, invasiveness, and physiological impact. The cVA-ECMO approach involves direct intraoperative cannulation of the right atrium and ascending aorta, allowing more effective venous drainage and antegrade aortic flow, despite the cost of greater invasiveness and bleeding risk. While these characteristics may theoretically favor hemodynamic stability, such advantages have not been conclusively demonstrated in lung transplantation, where left ventricular function is often preserved and pulsatility is commonly maintained even under full ECMO support. Accordingly, proposed physiological mechanisms should be interpreted with caution, and observed differences are more likely to reflect patient selection, transplant indication, and perioperative management rather than intrinsic hemodynamic superiority. In contrast, pVA-ECMO is typically established via femoral vessels, allowing rapid and less invasive deployment and preserving an unobstructed surgical field, but at the expense of retrograde arterial flow and a higher risk of flow- and access-related complications. In particular, peripheral cannulation is often related to differential hypoxemia, a phenomenon resulting from competing retrograde ECMO flow and native cardiac output, which may lead to inadequate oxygenation of the upper body despite satisfactory lower-body perfusion. As highlighted by Shigemura et al.,^31^ differences in flow direction and oxygenation patterns between central and peripheral configurations are especially relevant in lung transplantation and require vigilant monitoring of upper-body oxygenation. Consistent with current American Association for Thoracic Surgery recommendations,^32^ ECMO configuration should therefore be individualized according to patient characteristics and intraoperative requirements rather than applied uniformly.

In this context, our results demonstrate that although survival and PGD3 incidence are comparable between cVA-ECMO and pVA-ECMO, clinically meaningful differences emerge in postoperative morbidity and resource utilization. By incorporating studies published up to 2025 and applying reconstruction of individual patient survival data, this meta-analysis provides an updated synthesis of the literature, reinforcing the concept that ECMO configuration primarily influences early postoperative recovery rather than survival itself.^23^, ^24^, ^25^, ^26^, ^27^

Consistent with this interpretation, reconstructed survival analysis showed no significant difference in overall survival between cannulation strategies. Despite their distinct technical and hemodynamic characteristics, both cVA-ECMO and pVA-ECMO are capable of providing adequate hemodynamic and respiratory support. The physiological differences between antegrade and retrograde arterial flow, while relevant for some postoperative outcomes, appear insufficient to translate into measurable survival differences in this population.

Several postoperative outcomes differed between cannulation strategies. The need for postoperative ECMO and the duration of extracorporeal support were both significantly higher in the pVA-ECMO group. From a physiological rationale, this observation is consistent with the concept that central cannulation, by providing more effective venous drainage and antegrade aortic perfusion, may allow a smoother hemodynamic transition after graft implantation. Previous studies by Hoetzenecker et al.^5^ and Fernandez et al.^23^ have shown that retrograde femoral arterial flow can increase left ventricular afterload, impair ventricular unloading, and potentially prolong extracorporeal support. Nevertheless, these findings should be interpreted with caution. The observed differences are likely influenced, at least in part, by underlying transplant indications, particularly pulmonary hypertension, in which planned continuation of ECMO into the postoperative period is common and peripheral cannulation is frequently preferred. This confounding by indication limits causal inference and underscores that postoperative ECMO requirements may reflect patient-specific factors as much as the cannulation strategy itself.

Despite the absence of a significant difference in the incidence of PGD3, this outcome showed high heterogeneity, likely reflecting differences in institutional protocols and surgical techniques. In addition, pVA-ECMO was also associated with longer mechanical ventilation time and ICU LOS, suggesting delayed respiratory recovery and potential hemodynamic impact of the peripheral flow pattern.

The higher morbidity observed in pVA-ECMO group can be explained by pathophysiological mechanisms related to differences in arterial flow after graft implantation. Central VA-ECMO promotes anterograde flow in the ascending aorta, ensuring more physiological hemodynamic synchrony, more efficient right atrial drainage, and less left ventricular overload, which favors more homogeneous graft reperfusion and a lower risk of early pulmonary congestion.^5^, ^6^, ^7^, ^23^, ^28^ In contrast, pVA-ECMO generates retrograde flow from the femoral artery, increasing ventricular afterload and potentially compromising graft perfusion which may contribute to reperfusion edema, prolonged extracorporeal support, and extended mechanical ventilation.^5^, ^6^, ^7^, ^23^, ^27^, ^28^

From a safety standpoint, pVA-ECMO presented a higher risk of limb ischemia, a finding widely recognized in the literature. On the other hand, cVA-ECMO, although more invasive due to direct cannulation of the right atrium and aorta, was not associated with a higher incidence of intrathoracic complications, reinforcing its safety in experienced centers.

Sensitivity analysis highlighted the inherent fragility of the limited number of studies, with changes in the direction and magnitude of the effects when certain studies were excluded. The absence of detailed intraoperative data restricts the ability to identify more specific physiological mechanisms involved in the differences found.

This study has some limitations. First, all included studies were observational, introducing inherent risks of bias, particularly treatment allocation bias, as cannulation strategy and even ECMO initiation were frequently driven by intraoperative instability, intolerance to pulmonary artery clamping, hypoxemia, or ventricular dysfunction rather than by predefined criteria; consequently, patients requiring emergent or rescue ECMO likely represented a more severely ill subgroup. In addition, the limited number of available comparative studies reflects the current evidence base and restricts the scope of inference; while several single-center series have reported favorable outcomes with exclusive use of peripheral VA-ECMO, these studies lack an internal comparator and were therefore not eligible for inclusion. Substantial heterogeneity across several secondary outcomes further limits generalizability and likely reflects variability in patient selection, surgical technique, and perioperative management across centers. Third, the lack of standardized intraoperative and postoperative ECMO protocols among the included studies further limits the interpretation of results and precludes more detailed mechanistic analyses. Finally, surgical incision type was inconsistently reported, precluding adjustment for this potentially relevant intraoperative confounder.

Despite these limitations, this meta-analysis has important strengths. We conducted a comprehensive and methodologically rigorous systematic review following prespecified criteria and established standards. All available studies directly comparing central and peripheral VA-ECMO during lung transplantation were included, and risk of bias was systematically assessed using validated tools. Although the overall risk ranged from moderate to serious, the consistency of several associations across studies strengthens the robustness of our findings.

## Conclusion

Although survival and PGD3 incidence were comparable, pVA-ECMO was associated with greater postoperative morbidity and vascular complications. These findings should be interpreted with caution, and cannulation strategy should be individualized according to patient risk profile, surgical context, and center-specific expertise rather than favoring one approach uniformly.

## Declaration of Interest Statement

The authors declare that they have no known competing financial interests or personal relationships that could have appeared to influence the work reported in this paper.

## Funding

This work was supported by the Deutsche Forschungsgemeinschaft (DFG, German Research Foundation to Tulio Caldonazo) Clinician Scientist Program OrganAge funding number 413668513, by the Deutsche Herzstiftung (DHS, German Heart Foundation to Tulio Caldonazo) funding number S/03/23 and by the Interdisciplinary Center of Clinical Research of the Medical Faculty Jena.

## Data Availability Statement

The data underlying this article are available in the article and in its online supplementary material.

## Disclosures

None.

## Footnotes

Appendix A Supplemental data associated with this article can be found in the online version at doi:10.1016/j.jhlto.2026.100502.
