# Supplementary material for: Central versus peripheral veno-arterial extracorporeal membrane oxygenation during lung transplantation: a systematic review and meta-analysis
Source: JHLT Open. 2026 Jan 29;12:100502. doi: 10.1016/j.jhlto.2026.100502 (PMC12924189; doi:10.1016/j.jhlto.2026.100502)

**SUPPLEMENTARY MATERIAL**

**SUPPLEMENTARY TABLES AND FIGURES LEGENDS:**

**Supplementary Table 1.** Search strategy for each database.

**Supplementary Table 2.** Detailed reasons for excluding studies after full-text review.

**Supplementary Table 3.** Detailed donor characteristics across studies included in the meta-analysis.

**Supplementary Table 4.** Rationale for risk of bias assessment.

**Supplementary Figure 1.** Two-stage survival meta-analysis comparing peripheral versus central VA-ECMO during lung transplantation. HR = hazard ratio; CI = confidence interval; VA-ECMO = venoarterial extracorporeal membrane oxygenation.

**Supplementary Figure 2.** Leave-one-out sensitivity analysis for grade 3 primary graft dysfunction (PGD3) at 72 hours comparing peripheral versus central VA-ECMO. CI = confidence interval; OR = odds ratio; VA-ECMO = venoarterial extracorporeal membrane oxygenation.

**Supplementary Figure 3.** Leave-one-out sensitivity analysis for postoperative ECMO use comparing peripheral versus central VA-ECMO. CI = confidence interval; OR = odds ratio; VA-ECMO = venoarterial extracorporeal membrane oxygenation.

**Supplementary Figure 4.** Leave-one-out sensitivity analysis for postoperative ECMO duration comparing peripheral versus central VA-ECMO. CI = confidence interval; MD = mean difference; VA-ECMO = venoarterial extracorporeal membrane oxygenation.

**Supplementary Table 1.** Search strategy for each database.

| **PubMed** | ("Lung Transplantation"[Mesh] OR "lung transplantation"[tiab] OR "lung transplant"[tiab] OR "pulmonary transplant"[tiab])  **AND**  ("Extracorporeal Membrane Oxygenation"[Mesh] OR "extracorporeal membrane oxygenation"[tiab] OR ECMO[tiab] OR "VA-ECMO"[tiab] OR "veno-arterial ECMO"[tiab] OR "veno arterial extracorporeal membrane oxygenation"[tiab])  **AND**  ("central cannulation"[tiab] OR "central VA-ECMO"[tiab] OR "central arterial cannulation"[tiab] OR "central aortic cannulation"[tiab] OR "peripheral cannulation"[tiab] OR "peripheral VA-ECMO"[tiab] OR "peripheral arterial cannulation"[tiab] OR femoral[tiab] OR axillary[tiab] OR iliac[tiab]) |
| --- | --- |
| **Embase** | ('lung transplantation'/exp OR 'lung transplantation':ti,ab,kw OR 'lung transplant':ti,ab,kw OR 'pulmonary transplant':ti,ab,kw)  **AND**  ('extracorporeal membrane oxygenation'/exp  OR 'extracorporeal membrane oxygenation':ti,ab,kw  OR ECMO:ti,ab,kw  OR 'va-ecmo':ti,ab,kw  OR 'veno-arterial ecmo':ti,ab,kw  OR 'veno arterial extracorporeal membrane oxygenation':ti,ab,kw)  **AND**  ('central cannulation':ti,ab,kw  OR 'central va-ecmo':ti,ab,kw  OR 'central arterial cannulation':ti,ab,kw  OR 'central aortic cannulation':ti,ab,kw  OR 'peripheral cannulation':ti,ab,kw  OR 'peripheral va-ecmo':ti,ab,kw  OR 'peripheral arterial cannulation':ti,ab,kw  OR femoral:ti,ab,kw  OR axillary:ti,ab,kw  OR iliac:ti,ab,kw) |
| **Cochrane** | ("lung transplant" OR "lung transplantation")  **AND**  (ECMO OR "extracorporeal membrane oxygenation")  **AND**  (cannulation OR central OR peripheral OR femoral OR axillary) |

**Supplementary Table 2.** Detailed reasons for excluding studies after full-text review.

| **First author, year** | **Title** | **DOI** | **Reason for exclusion** |
| --- | --- | --- | --- |
| Patrick, 2021 | Mobilization of Patients Receiving Extracorporeal Membrane Oxygenation Before Lung Transplant. | 10.4037/ccn2021689 | Wrong intervention, wrong comparator, wrong timing, not VA-ECMO |
| Schoeberl, 2024 | Alternative venous access sites for dual-lumen extracorporeal membrane oxygenation cannulation. | 10.1093/icvts/ivae060 | Wrong ECMO type (VV, not VA) + wrong timing (not intraoperative) + wrong comparator (no central vs peripheral VA-ECMO) |
| Toubat, 2025 | Current intraoperative mechanical circulatory support strategies for bilateral lung transplantation surgery. | 10.1016/j.xjtc.2025.02.020 | Narrative review / technical algorithm article |

**Supplementary Table 3.** Detailed donor characteristics across studies included in the meta-analysis.

| First author, year | Donor Age, years | | Male Donor Sex, n (%) | | Donor Ever Smoked, n (%) | | Donor Circulatory Death, (%) | | Ventilation, days | |
| --- | --- | --- | --- | --- | --- | --- | --- | --- | --- | --- |
|  |  |  |  |  |  |  |  |  |  |  |
|  | Peripheral | Central | Peripheral | Central | Peripheral | Central | Peripheral | Central | Peripheral | Central |
| Fernandez, 2025 | 41.83 ± 14.90* | 39.48 ± 15.37* | 101 (54.3) | 123 (39.0) | 73 (40.8) | 139 (45.0) | 65 (34.9) | 97 (30.8) | NA | NA |
| Glorion, 2018 | 51 (41–39) | 46.5 (35–55) | 24 (45) | 22 (45) | 25 (51) | 27 (50) | NA | NA | 2 (1–3) | 2 (1–3) |
| Li, 2024 | NA | NA | NA | NA | NA | NA | NA | NA | NA | NA |
| Ruszel, 2021 | NA | NA | NA | NA | NA | NA | NA | NA | NA | NA |
| Wu, 2025 | 38.0 ± 11.8* | 37.2 ± 12.1* | 57 (62.6) | 23 (74.2) | NA | NA | 9 (9.9) | 4 (12.9) | 4.0 (3–7) | 3.8 (3–6.5) |
| Continuous data are provided in median (IQR), unless indicated otherwise. NA: not available. | | | | | | | | | | |

**Supplementary Table 4.** Rationale for risk of bias assessment.

| Study | D1 Confounding | D2 Selection | D3 Classification | D4 Deviations | D5 Missing Data | D6 Measurement | D7 Reporting |
| --- | --- | --- | --- | --- | --- | --- | --- |
| Fernandez 2025 | Moderate: Retrospective design allows residual confounding, though groups appear reasonably comparable. | Moderate: Some exclusions and clinical criteria may influence allocation. | Low: ECMO modality clearly defined and recorded. | Low: No deviations related to intervention. | Moderate: Some variables missing but key outcomes complete. | Low: PGD and major outcomes objectively assessed. | Moderate: No preregistered protocol; selective reporting possible. |
| Glorion 2018 | Moderate: Clinical status may influence ECMO type, but baseline characteristics are well described. | Moderate: Decision-making reflects real-world practice; some selection possible. | Low: ECMO type documented clearly. | Low: No deviations identified. | Low: Minimal missing data. | Moderate: Some complications partially subjective. | Moderate: No protocol; major outcomes fully reported. |
| Li 2025 | Moderate: Non-randomized design may introduce imbalances, though groups show reasonable comparability. | Moderate: Consecutive inclusion with some clinical selection influence. | Low: Interventions well defined. | Low: No deviations. | Moderate: Some perioperative data missing, but outcomes complete. | Moderate: Some subjective outcomes without blinding. | Moderate: Reporting comprehensive despite no protocol. |
| Ruszel 2021 | Moderate: Allocation influenced by intraoperative condition; baseline described adequately. | Moderate: Selection driven by clinical response but consistent with practice. | Low: Classification objective (CPB, pECMO, cECMO). | Low: No deviations described. | Moderate: Missingness not fully described. | Moderate: Some outcomes partially subjective. | Moderate: No protocol; selective reporting cannot be excluded. |
| Wu 2025 | Moderate: Non-randomized design allows moderate confounding; groups largely comparable. | Moderate: Consecutive inclusion; modality influenced by intraoperative findings. | Low: ECMO modality clearly defined and consistently applied. | Low: No deviations between groups. | Low: Very limited missing data. | Moderate: Some outcomes dependent on clinical judgment. | Moderate: No preregistration, but outcomes well reported. |

**Supplementary Figure 1.** Two-stage survival meta-analysis comparing peripheral versus central VA-ECMO during lung transplantation. HR = hazard ratio; CI = confidence interval; VA-ECMO = venoarterial extracorporeal membrane oxygenation.


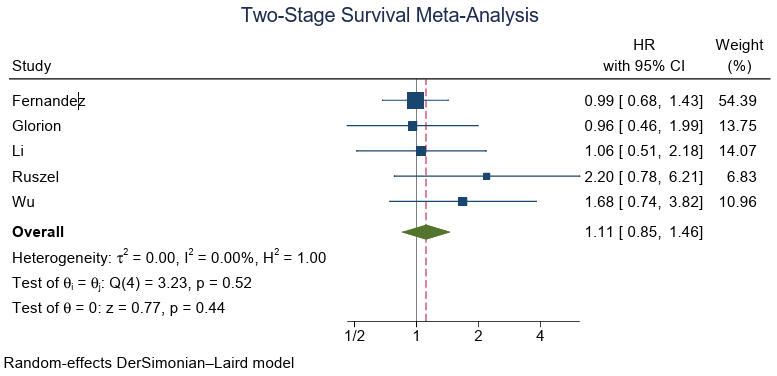


**Supplementary Figure 2.** Leave-one-out sensitivity analysis for grade 3 primary graft dysfunction (PGD3) at 72 hours comparing peripheral versus central VA-ECMO. CI = confidence interval; OR = odds ratio; VA-ECMO = venoarterial extracorporeal membrane oxygenation.


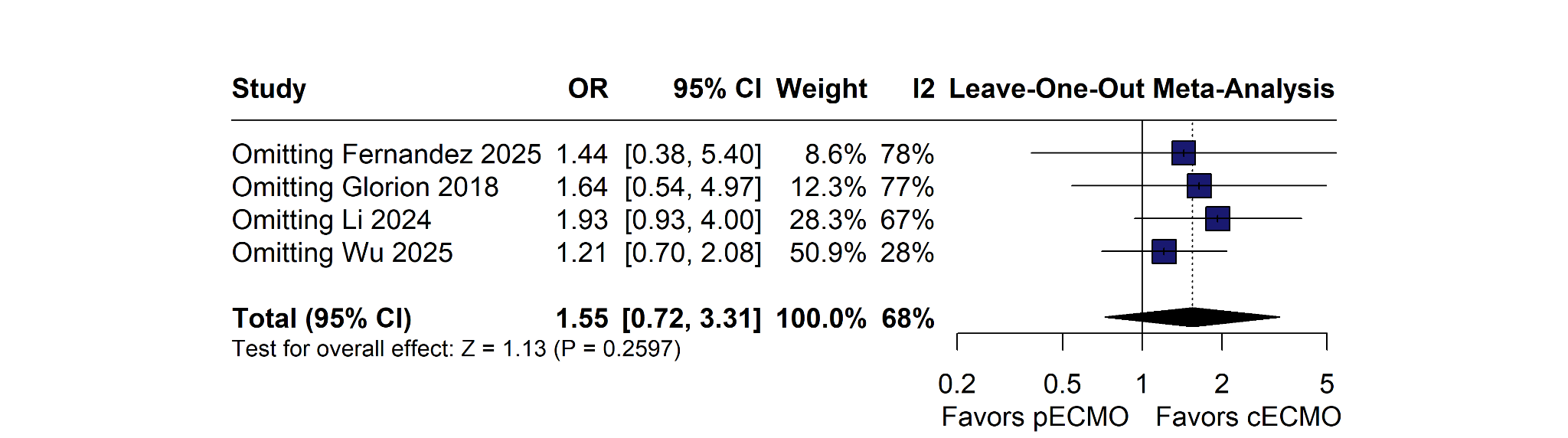


**Supplementary Figure 3.** Leave-one-out sensitivity analysis for postoperative ECMO use comparing peripheral versus central VA-ECMO. CI = confidence interval; OR = odds ratio; VA-ECMO = venoarterial extracorporeal membrane oxygenation.


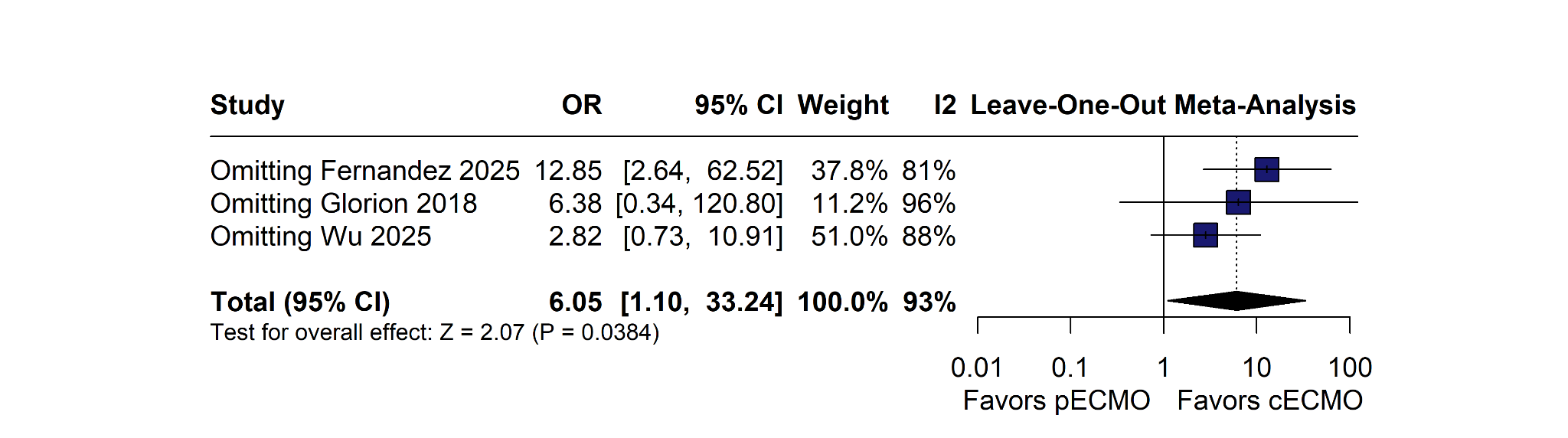


**Supplementary Figure 4.** Leave-one-out sensitivity analysis for postoperative ECMO duration comparing peripheral versus central VA-ECMO. CI = confidence interval; MD = mean difference; VA-ECMO = venoarterial extracorporeal membrane oxygenation.


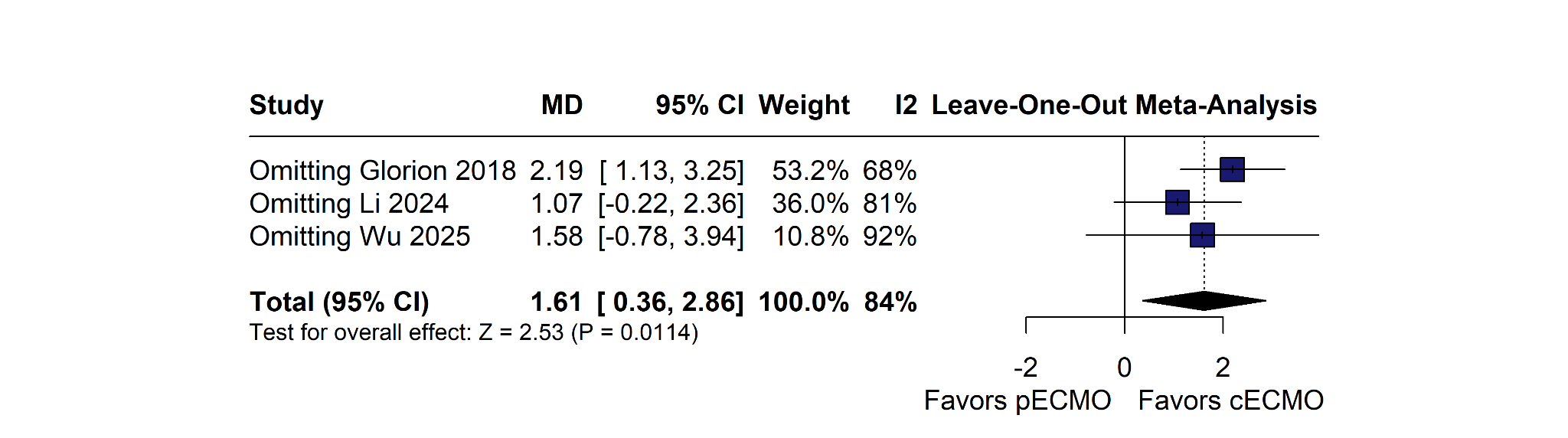

Supplement: Supplementary file 1 — Supplemental material [file mmc1.docx]
